# Liberal versus restrictive red blood cell transfusion strategy in sepsis or septic shock: a systematic review and meta-analysis of randomized trials

**DOI:** 10.1186/s13054-019-2543-1

**Published:** 2019-07-25

**Authors:** Yohei Hirano, Yukari Miyoshi, Yutaka Kondo, Ken Okamoto, Hiroshi Tanaka

**Affiliations:** Department of Emergency and Critical Care Medicine, Juntendo University Urayasu Hospital, 2-1-1 Tomioka, Urayasu, Chiba 279-0021 Japan

**Keywords:** Sepsis, Septic shock, Transfusion, Hemoglobin, Threshold, Mortality

## Abstract

**Background:**

We assessed the effect of liberal versus restrictive red blood cell transfusion strategy on survival outcome in sepsis or septic shock by systematically reviewing the literature and synthesizing evidence from randomized controlled trials (RCTs).

**Methods:**

We searched the MEDLINE, Cochrane Central Register of Controlled Trials, and Web of Science databases. We included RCTs that compared mortality between a liberal transfusion strategy with a hemoglobin threshold of 9 or 10 g/dL and a restrictive transfusion strategy with a hemoglobin threshold of 7 g/dL in adults with sepsis or septic shock. Two investigators independently screened citations and conducted data extraction. The primary outcome was 28- or 30-day mortality. Secondary outcomes were 60- and 90-day mortality, use of life support at 28 days of admission, and number of patients transfused during their intensive care unit stay. DerSimonian-Laird random-effects models were used to report pooled odds ratios (ORs).

**Results:**

A total of 1516 patients from three RCTs were included; 749 were randomly assigned to the liberal transfusion group and 767 to the restrictive strategy group. Within 28–30 days, 273 patients (36.4%) died in the liberal transfusion group, while 278 (36.2%) died in the restrictive transfusion group (pooled OR, 0.99; 95% confidence interval [CI], 0.67–1.46). For the primary outcome, heterogeneity was observed among the studies (*I*^2^ = 61.0%, *χ*^2^ = 5.13, *p* = 0.08). For secondary outcomes, only two RCTs were included. There were no significant differences in secondary outcomes between the two groups.

**Conclusions:**

We could not show any difference in 28- or 30-day mortality between the liberal and restrictive transfusion strategies in sepsis or septic shock patients by meta-analysis of RCTs. Our results should be interpreted with caution due to the existence of heterogeneity. As sepsis complicates a potentially wide range of underlying diseases, further trials in carefully selected populations are anticipated.

**Trial registration:**

This present study was registered in the PROSPERO database (CRD42018108578).

**Electronic supplementary material:**

The online version of this article (10.1186/s13054-019-2543-1) contains supplementary material, which is available to authorized users.

## Background

Patients with sepsis or septic shock are often anemic and undergo red blood cell (RBC) transfusion. This intervention may restore the balance between oxygen supply and demand by increasing oxygen delivery into tissue [1, 2]. However, RBC transfusion may also lead to deleterious events such as cardiovascular overload, acute kidney injury, acute lung injury, infectious complications, and immunomodulation [3–5]. With these adverse effects of RBC transfusion, clinicians are challenged with selecting the optimal threshold of transfusion for sepsis patients to improve survival outcome.

Two different strategies of RBC transfusion (“liberal” transfusion with a hemoglobin threshold of 9 or 10 g/dL and “restrictive” transfusion with a hemoglobin threshold of 7 g/dL) have been compared in observational studies and a few randomized controlled trials (RCTs) to assess which strategy would be more beneficial in treating sepsis patients [6]. After the introduction of the systematic review process, the newest international guidelines for the management of sepsis and septic shock (Surviving Sepsis Campaign, 2016) recommended restrictive RBC transfusions only when the hemoglobin level decreases to < 7.0 g/dL in adult sepsis in the absence of extenuating circumstances, such as myocardial ischemia, severe hypoxemia, or acute hemorrhage [7]. However, this recommendation is based on limited data from only two clinical trials, including one less-direct assessment of blood transfusion therapy [8, 9].

Until now, there has been no meta-analysis reviewing only RCTs on hemoglobin threshold for RBC transfusion in sepsis. Therefore, we aimed to conduct a systematic review and meta-analysis of present RCTs to assess the effect of liberal versus restrictive RBC transfusion strategy on survival outcome in sepsis or septic shock patients.

## Methods

### Data sources and search strategies

To identify eligible trials, we searched the Cochrane Central Register of Controlled Trials, the MEDLINE, and the Web of Science databases on June 21, 2018. Searches were not restricted by publication status, publication date, and sample size. We did not search references of articles. The search terms used were “(sepsis OR septic shock) AND (transfusion OR transfused OR transfusions OR hemoglobin) AND (randomised OR randomized)”.

### Study selection

Titles and abstracts of references were retrieved from the databases. After all duplicate studies were excluded, two investigators (YH and YM) independently screened the titles and abstracts for eligibility. When a disagreement was identified between reviewers, the full text of the article was obtained to determine the study’s eligibility, and differences in opinion were resolved by consensus. If disagreements could not be reconciled, a third investigator (YK) was consulted. The full texts of articles included in the final selection were independently reviewed by two investigators (YH and YM). Finally, eligible studies were determined after discussion and resolution of discrepancies by consensus.

We identified the studies to be included by following a research question formulated according to the participants, interventions, comparisons, and outcomes (PICO) model, as follows: P, adult (≥ 18 year old) patients admitted to the intensive care unit (ICU) with a diagnosis of sepsis or septic shock; I, liberal RBC transfusion strategy (blood transfusion with a hemoglobin threshold of ≤ 9–10 g/dL); C, restrictive RBC transfusion strategy (blood transfusion with a hemoglobin threshold of ≤ 7 g/dL; and O, all-cause mortality. We did not restrict the definition of sepsis to the latest definition [10]; instead, we allowed all past definitions of sepsis when identifying the studies to be included.

### Data extraction

Data were extracted independently by two investigators, and consensus was reached. The data extracted included the following: author, publication year, country, study design, number and type of participants, severity and source of sepsis, timing of randomization, duration of intervention, trial exclusion criteria, inclusion period, hemoglobin threshold, leukodepletion of blood transfused, outcome measures, and study results.

### Study endpoints

We set short-term mortality defined as 28- or 30-day mortality as the primary outcome. The secondary outcomes were 60- and 90-day mortality, use of life support (ventilation, vasopressor use, or renal replacement therapy [RRT]) at 28 days of admission, and number of patients transfused during their ICU stay.

#### Assessment of methodological quality

We adapted the Cochrane risk-of-bias tool to assess the quality of the studies included for meta-analysis [11]. Two investigators (YH and YM) independently assessed the risk of bias of the included studies, and a third investigator (YK) resolved the discrepancies using an independent blinded evaluation. Additionally, we graded the quality of evidence of each finding based on the criteria established by the Grading of Recommendations Assessment, Development and Evaluation (GRADE) working group [12]. The quality of the study methodology was independently classified by the two investigators as high, intermediate, low, or very low, based on study design, risk of bias, indirectness, inconsistency, imprecision, and publication bias. The publication biases were assessed visually by inspecting funnel plots as well as analytical appraisals based on Egger’s linear regression test [13]. A two-sided *p* value of ≤ 0.10 was regarded as significant in Egger’s linear regression test.

#### Statistical analysis

We pooled the eligible patients for each outcome and calculated the odds ratios (ORs) and corresponding 95% confidence intervals (CIs) using the DerSimonian-Laird random-effects model with weights calculated using the inverse variance method. We verified the heterogeneity of the studies using the Cochran chi-squared, tau-squared, and *I*^2^ statistics (*I*^2^ > 50% was considered a measure of severe heterogeneity). We applied unadjusted *p* values for the significance assessment in this study, which were set at the two-tailed 0.05 level for hypothesis testing and at the 0.10 level for heterogeneity testing. All statistical analyses were performed using the Cochrane systematic review software Review Manger version 5.3.5 for Windows (The Nordic Cochrane Centre, the Cochrane Collaboration, Copenhagen, Denmark), except for the analysis of publication bias, which was through Stata version 15SE^®^ (StataCorp LP, 2013).

## Results

### Search results

**Fig. 1:**
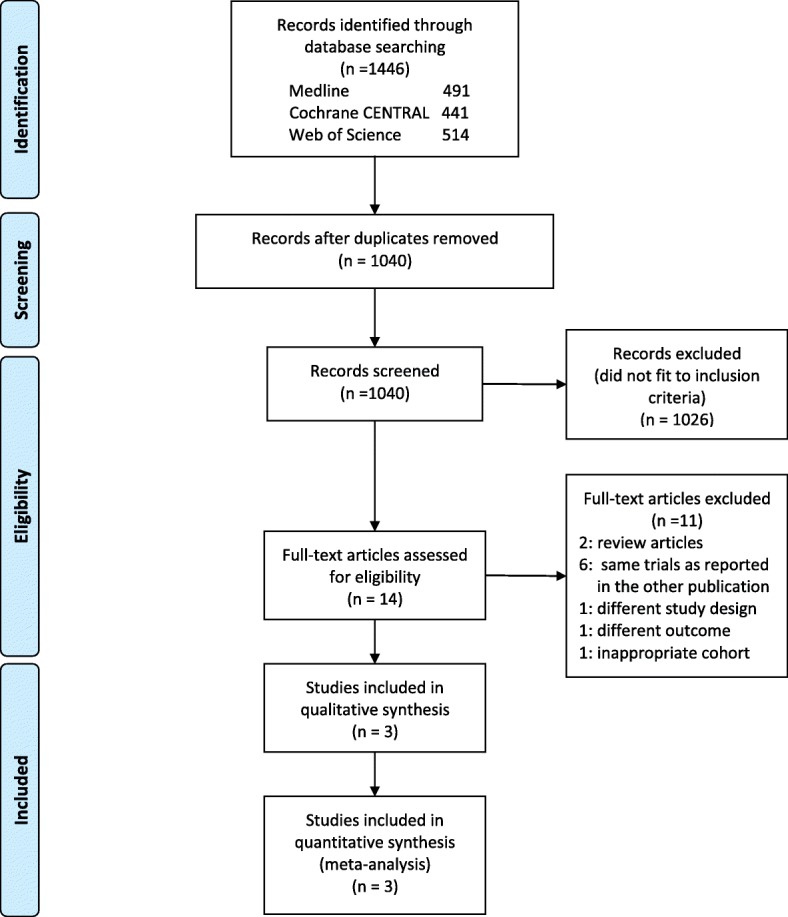
Flow diagram of search strategy and study selection

We identified 1040 studies from the electronic databases after elimination of duplicates. Among them, only 14 studies were eligible based on the assessment of the title and abstract. After review of their full-text articles, 11 studies were excluded because they were reviews or the same trials reported in the other studies included, they were conducted with a different study design or outcome, or they involved an inappropriate cohort. Finally, three RCTs were included in this meta-analysis [8, 14, 15] (Fig. 1).

### Study characteristics

We analyzed a total of 1516 patients from the three RCTs, namely TRICOP, TRISS, and TRICC. Among them, 749 patients were randomly assigned to the liberal transfusion group and 767 to the restrictive transfusion group. Two of the three studies were multicentric studies (TRISS and TRICC). Participants in the TRISS study were patients with septic shock, while those in the TRICOP study were cancer patients with septic shock. In the TRICC study, the participants were critically ill patients and not restricted to sepsis patients. However, a subgroup analysis was performed for patients with severe infection or septic shock. Although lungs were the most frequent cause of sepsis in the TRICOP and TRISS trials, there was a diversity in the source of infection among studies. All trials excluded patients with uncontrolled bleeding or the withdrawal from active therapy. However, there were a variety in exclusion criteria among studies, represented by acute myocardial ischemia which was only excluded in the TRISS trial. In all studies, RBCs were transfused when the hemoglobin level was < 7 g/dL for restrictive strategy. The hemoglobin threshold for liberal transfusion was set at < 9 g/dL in the TRISS and TRICOP trials, and at < 10 g/dL in the TRICC trial. All RBC units were leukodepleted in two of the three studies (TRICOP and TRISS). The individual characteristics of the three RCTs are detailed in Table 1.

**Table 1:** Detail of included studies

	TRICOP	TRISS	TRICC
First author, year	Bergamin, 2017 [14]	Holst, 2014 [8]	Hebert, 1999 [15]
Country	Brazil	Denmark, Sweden, Norway Finland	Canada
No of site	1	32	25
Inclusion period	2012–2014	2011–2013	1994–1997
No of patients	300	998	218 (subgroup)
Type of patients	Cancer patients with septic shock (≧ 18 years old)	Patients with septic shock (≧ 18 years old)	Patients with severe infection or septic shock (≧ 16 years old)
Source of infection [liberal (%) vs restrictive (%)]			
Lungs	69 vs 61	52.2 vs 53.2	
Abdomen	13 vs 17	39.9 vs 41.0	Unknown
Urinary tract	9 vs 5	12.3 vs 11.6	
Other	9 vs 17	21.4 vs 21.8	
Exclusion criteria (yes: excluded, no: not excluded)			
Life-threating/uncontrolled bleeding	Yes	Yes	Yes
Acute myocardial ischemia	No	Yes	No
Admission after cardiac surgery	No	No	Yes
Hematologic cancer	Yes	No	No
Acute burn injury	No	Yes	No
End-stage renal disease	Yes	No	No
Pregnancy	No	No	Yes
Anticoagulation therapy	Yes	No	No
Withdrawal from active therapy	Yes	Yes	Yes
Hemoglobin threshold [liberal (g/dL) vs restrictive (g/dL)]	< 9 vs < 7	< 9 vs < 7	< 10 vs < 7
Severity of patients (SOFA score) [median (interquartile range)]	Liberal; 6 (5–9)	Liberal; 10 (8–12)	Unknown
	Restrictive; 7 (5–9)	Restrictive; 10 (8–12)	
Timing of randomization after ICU admission (h) [median (interquartile range)]	Within 6 h	Liberal; 20 (7–43)	Within 72 h
		Restrictive; 23 (7–50)	
Intervention period	ICU stay	ICU stay	ICU stay
Leukodepletion	Yes	Yes	No
Outcomes	Mortality (28, 60, 90 days)	Mortality (28, 60, 90 days)	Mortality (30 days)
	Ischemic events	Ischemic events	
	Severe adverse reactions	Severe adverse reactions	
	Use of life support at 28 days	Use of life support at 28 days	
	Number of patients transfused in the ICU	Number of patients transfused in the ICU	

### Outcome

**Fig. 2:**
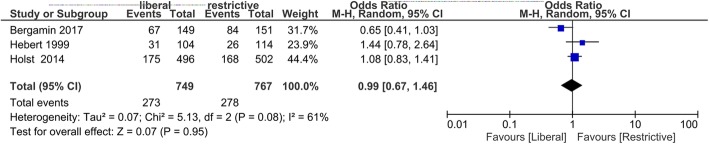
Forest plot of the 28- or 30-day mortality compared between liberal and restrictive blood transfusion strategy in sepsis or septic shock

**Fig. 3:**
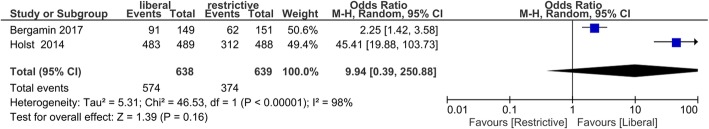
Forest plot of the number of patients transfused with RBC in the ICU in comparison between liberal and restrictive blood transfusion strategy

The forest plot of the primary outcome is shown in Fig. 2. Within 28–30 hospital days, 273 of 749 patients (36.4%) died in the liberal transfusion group, while 278 of 767 patients (36.2%) died in the restrictive transfusion group. There was no difference in 28- or 30-day mortality between the two transfusion strategies (pooled OR, 0.99; 95% CI, 0.67–1.46). In the evaluation of secondary outcomes, only two RCTs (TRICOP and TRISS) were included because we could not obtain any information about the outcomes other than 30-day mortality from the subgroup analysis of patients with severe infection or septic shock in the TRICC study. Similar to 28- or 30-day mortality, there were no significant differences in 60- or 90-day mortality between the liberal and restrictive transfusion strategies (pooled OR, 0.91 [95% CI, 0.55–1.51] or 0.85 [95% CI, 0.49–1.47], respectively) (Additional files 1 and 2). The number of patients transfused with RBCs in the ICU tended to be higher in the liberal transfusion group than in the restrictive transfusion group (pooled OR, 9.94; 95% CI, 0.39–250.88), although there was no significant difference, possibly due to wide distribution (Fig. 3). Moreover, there were no significant differences in the use of life support (ventilation, vasopressor use, or RRT) at 28 days of admission and the number of patients whom FFP or platelets were transfused during ICU stay between the two groups (Additional files 3, 4, 5, 6, and 7).

### Heterogeneity

For the primary outcome of 28- or 30-day mortality, heterogeneity was observed among the studies (*I*^2^ = 61.0%, *χ*^2^ = 5.13, *p* = 0.08) (Fig. 2). The evaluation of heterogeneity for other outcomes was described in forest plots (Fig. 3, Additional files 1, 2, 3, 4, 5, 6, and 7).

### Publication bias, risk of bias, and quality of evidence

**Fig. 4:**
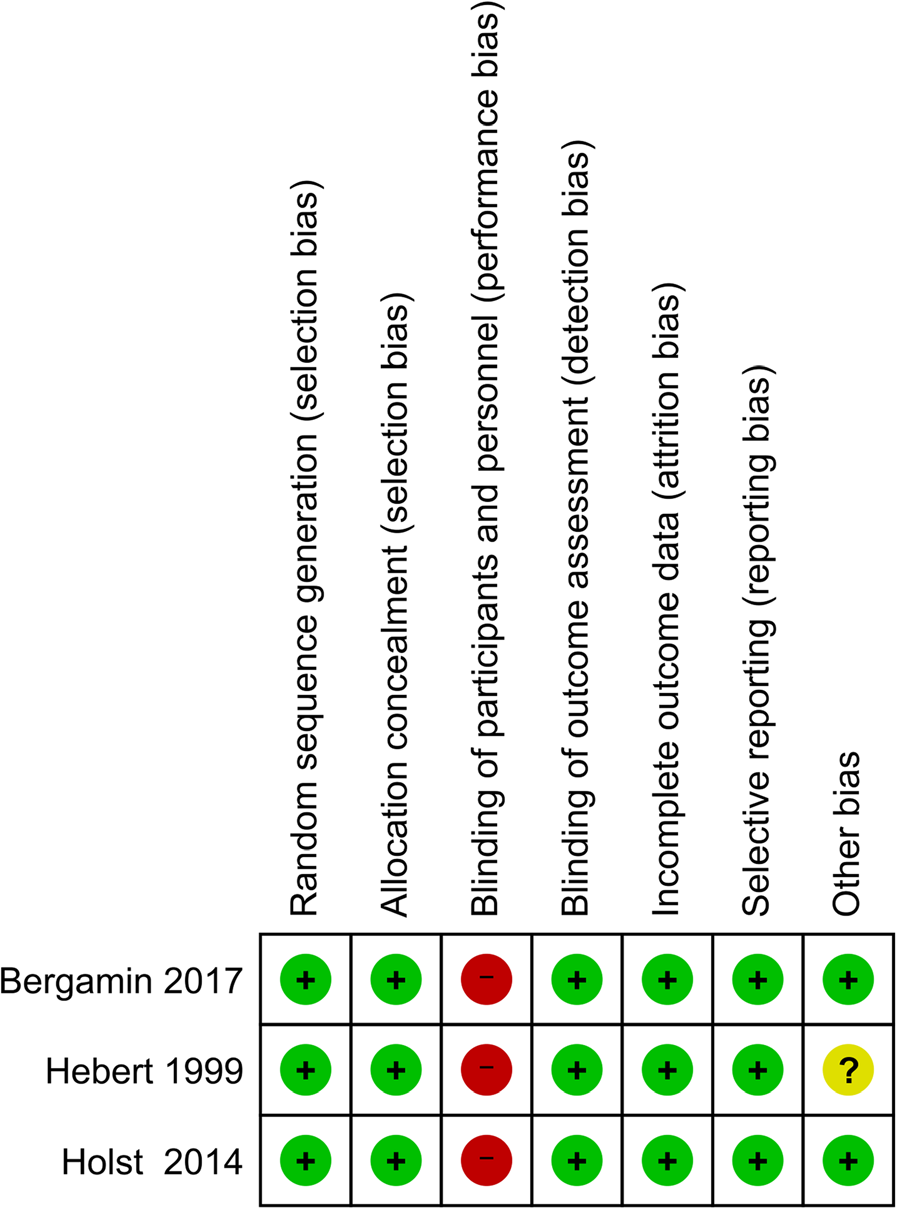
Risk of bias summary

**Fig. 5:**
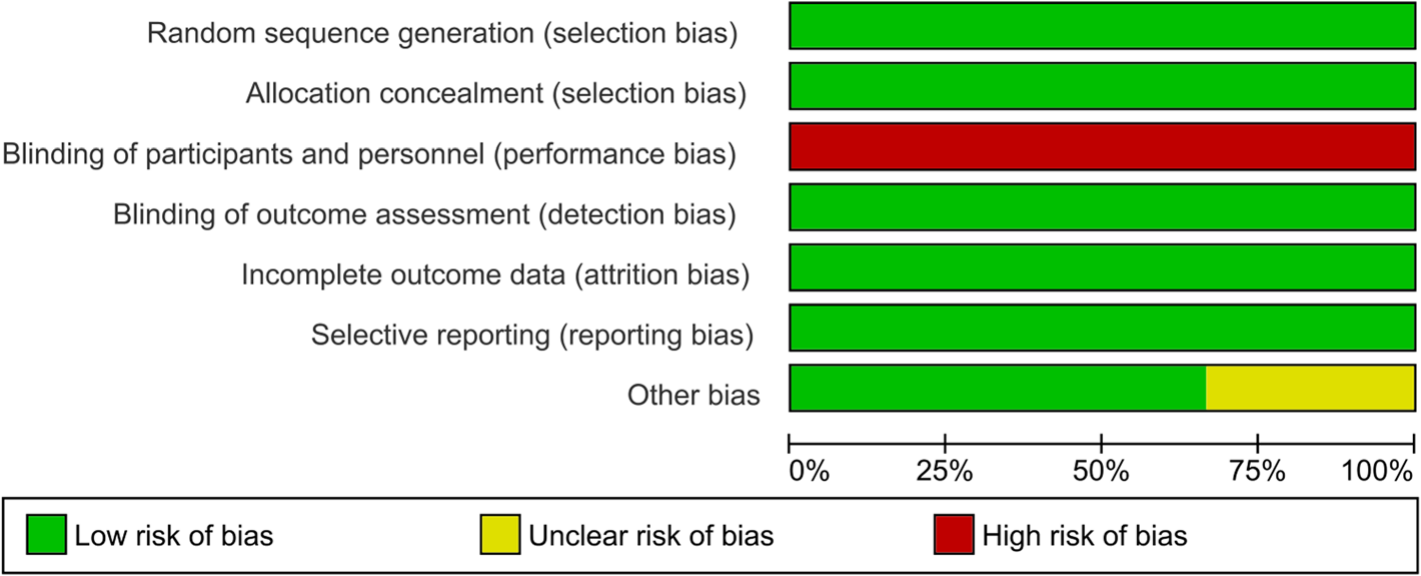
Risk of bias graph

We also analyzed the presence of publication bias for the primary outcome. A visual inspection of the funnel plot and Egger’s linear regression test showed no existence of publication bias in 28- or 30-day mortality (*p* = 0.294). Regarding risk of bias, the blinding of participants and personnel was categorized as high risk in all three RCTs due to the nature of intervention (Figs. 4 and 5). For the effect of liberal versus restrictive blood transfusion strategy on the primary outcome, the quality of evidence was rated as low. The grade was lowered by 2 points due to a major inconsistency in heterogeneity and indirectness of the studies such as presence of different populations of patients. The summary of evidences is detailed in Table 2.

**Table 2:** Summary of findings

Outcomes	Anticipated absolute effects^*^ (95% CI)		Relative effect: OR (95% CI)	No. of participants (no. of studies)	Certainty of evidence (GRADE)
	Risk with restrictive RBC transfusion	Risk with liberal RBC transfusion			
28- or 30-day mortality	362 per 1000	360 per 1000 (276–454)	0.99 (0.67–1.46)	1516 (3 RCTs)	Low^a,b^
60-day mortality	452 per 1000	429 per 1000 (312–554)	0.91 (0.55–1.51)	1298 (2 RCTs)	Low^b,c^
90-day mortality	493 per 1000	453 per 1000 (323–588)	0.85 (0.49–1.47)	1298 (2 RCTs)	Low^b,d^
No. of patients transfused with RBC in the ICU	585 per 1000	933 per 1000 (355–997)	9.94 (0.39–250.88)	1277 (2 RCTs)	Low^b,e^
Ventilation use at 28 days	146 per 1000	147 per 1000 (81–250)	1.01 (0.52–1.96)	1277 (2 RCTs)	Low^b,f^
Vasopressor use at 28 days	66 per 1000	69 per 1000 (28–161)	1.06 (0.41–2.72)	1277 (2 RCTs)	Low^b,g^
RRT at 28 days	66 per 1000	64 per 1000 (41–99)	0.97 (0.60–1.57)	1277 (2 RCTs)	Moderate^b^
No. of patients transfused with FFP in the ICU	186 per 1000	205 per 1000 (163–257)	1.13 (0.85–1.51)	1277 (2 RCTs)	Moderate^b^
No. of patients transfused with platelet in the ICU	146 per 1000	165 per 1000 (120–221)	1.16 (0.80–1.67)	1277 (2 RCTs)	Moderate^b^

Grades of evidence according to the Grading of Recommendations Assessment, Development and Evaluation (GRADE) working group

High certainty: We are very confident that the true effect lies close to that of the estimate of the effect

Moderate certainty: We are moderately confident in the effect estimate. The true effect is likely to be close to the estimate of the effect, but there is a possibility that it is substantially different

Low certainty: Our confidence in the effect estimate is limited: The true effect may be substantially different from the estimate of the effect

Very low certainty: We have very little confidence in the effect estimate. The true effect is likely to be substantially different from the estimate of the effect

*The risk in the intervention group (and its 95% confidence interval [CI]) was based on the assumed risk in the comparison group and the relative effect of the intervention (and its 95% CI)

^a^Heterogeneity was observed among the studies (*I*^2^ = 61.0%, *χ*^2^ = 5.13, *p* = 0.08). Downgraded by 1

^b^Indirectness such as different populations of patients was observed in the studies. Downgraded by 1

^c^Heterogeneity was observed among the studies (*I*^2^ = 72.0%, *χ*^2^ = 3.63, *p* = 0.06). Downgraded by 1

^d^Heterogeneity was observed among the studies (*I*^2^ = 77.0%, *χ*^2^ = 4.27, *p* = 0.04.) Downgraded by 1

^e^Heterogeneity was observed among the studies (*I*^2^ = 98.0%, *χ*^2^ = 46.53, *p* < 0.01). Downgraded by 1

^f^Heterogeneity was observed among the studies (*I*^2^ = 75.0%, *χ*^2^ = 4.04, *p* = 0.04). Downgraded by 1

^g^Heterogeneity was observed among the studies (*I*^2^ = 71.0%, *χ*^2^ = 3.49, *p* = 0.06). Downgraded by 1

*RBC* red blood cell, *OR* odds ratio, *RCT* randomized controlled trial, *ICU* intensive care unit, *RRT* renal replacement therapy, *FFP* fresh frozen plasma

## Discussion

To our knowledge, this study is the first systematic review and meta-analysis of RCTs investigating the effect of liberal versus restrictive RBC transfusion strategies on mortality in sepsis or septic shock patients. Although this meta-analysis included only three RCTs, the results suggested that the liberal strategy of RBC transfusion (with a hemoglobin threshold of 9 or 10 g/dL) failed to show any improvement in short-term (28- or 30-day) mortality in sepsis or septic shock patients, compared to the restrictive strategy (with a hemoglobin threshold of 7 g/dL).

Several studies have been conducted to determine the optimal blood transfusion threshold that would be the most beneficial to patient outcomes [16]. Hebert et al. conducted an RCT involving critically ill patients to compare the 30-day mortality rate between the liberal transfusion group (with a hemoglobin threshold of 10 g/dL) and restrictive transfusion group (with a hemoglobin threshold of 7 g/dL) (TRICC trial) [15]. They found no significant difference in outcome between the two strategies and, thus, recommended the use of the restrictive transfusion strategy in critically ill patients. However, the subgroup analysis of this study also raised the possibility that the suggested blood transfusion threshold could differ due to different diseases and patient populations. In fact, the very recent 2018 Frankfurt Consensus Conference on patient blood management strongly recommended the use of the restrictive RBC transfusion threshold in critically ill but clinically stable intensive care patients and in patients undergoing cardiac surgery; however, use of the restrictive RBC transfusion threshold was not clearly recommended in patients with hip fracture and cardiovascular diseases or other risk factors as well as in hemodynamically stable patients with acute gastrointestinal bleeding (conditional recommendation) [17]. Apparently, recent clinical trials investigating the hemoglobin threshold for transfusion have been more focused on narrow and specific populations [18–22].

Numerous cohort studies have been conducted on blood transfusion thresholds in patients with sepsis or septic shock. A systematic review of 12 cohort studies, including 9 studies focusing on mortality rates, has confirmed the safety of restrictive transfusion [6]. However, no meta-analysis has been performed of randomized trials on the hemoglobin threshold for RBC transfusion in this population due to the lack of RCTs. Similar to the abovementioned systematic review of cohort studies, our meta-analysis of RCTs revealed no differences in clinically important outcomes, such as 28- or 30-day mortality, between the liberal and restrictive blood transfusion strategies. Furthermore, we could not show any differences in secondary outcomes, such as 60- or 90-day mortality, use of life support (ventilation, vasopressor use, or RRT) at 28 days of admission, and number of patients who underwent FFP or platelet transfusion during ICU stay between the two strategies; however, only two RCTs were included for the analysis of secondary outcomes. Considering the limited availability of blood supply for transfusions, we recommend the use of the restrictive transfusion strategy over the liberal transfusion strategy in sepsis or septic shock patients.

Caution is required while interpreting our findings. The inclusion criteria for sepsis patients in each RCT analyzed were diverse. Particularly, the participants of the most recent RCT (TRICOP) were cancer patients with septic shock. In fact, the mortality rate was higher in this single-centered trial than in the other two trials. This study showed opposing results with respect to primary outcome, with a lower 28-day mortality rate in the liberal transfusion group, implying that the threshold of RBC blood transfusion in sepsis patients could differ according to the underlying medical conditions or diseases. Moreover, exclusion criteria also differed among the three RCTs. For example, acute myocardial ischemia patients were excluded from the TRISS trial but not from the TRICOP and TRICC trials, even though use of a restrictive transfusion threshold of < 80 g/L may not be safe in patients with underlying acute coronary syndrome or chronic cardiovascular disease [23]. As sepsis patients often show several complications, the patient’s background should be carefully considered while selecting between the liberal and restrictive strategies for blood transfusion. This heterogeneity in the comorbidities of sepsis patients also presents problems in transfusion trials where fixed interventions can cause practice misalignment in diverse populations. Further sepsis-related trials in carefully selected populations are anticipated.

### Limitations of the study

This meta-analysis has several limitations. First, only three RCTs were analyzed for the primary outcome and only two RCTs were analyzed for secondary outcomes. However, meta-analyses of too many low-quality studies can also cause bias. Thus, well-designed RCTs are required in the future to support our findings. Second, the participants and healthcare staff were aware of the group assignment in all included RCTs, resulting in potential performance bias. It was impossible to conceal the group assignment because of the characteristics of the intervention. However, this bias is unlikely to affect the results due to the use of stratified randomization and objective endpoints as well as the use of multicentered participants in two of the three RCTs. Third, the leukodepletion status of RBCs before transfusion was different among trials. As leukodepletion of blood products could reduce several complications of transfusion, such as alloimmunization of human leukocyte antigen (HLA), cytomegalovirus transmission, and recurrent febrile nonhemolytic transfusion reaction, this process could influence the outcome [24]. Finally, we observed some heterogeneities among the included trials, such as timing of randomization, inclusion and exclusion criteria, and source of sepsis. Even though systematic reviews and meta-analyses are considered to be powerful tools for solving research questions, heterogeneity is the one of the weaknesses of these statistical methods [25, 26].

## Conclusions

Based on the findings of this meta-analysis, we could not show any difference in short-term (28- or 30-day) mortality between the liberal RBC transfusion strategy with a hemoglobin threshold of 9 or 10 g/dL and the restrictive strategy with a hemoglobin threshold of 7 g/dL in sepsis or septic shock patients. We therefore recommend the restrictive transfusion strategy from the perspective of cost and resource saving; however, coexisting diseases such as cancer or cardiovascular disease in sepsis patients should be carefully considered. “Sepsis” or “septic shock” might still be too large as a category in deciding the best hemoglobin threshold for blood transfusion.

## Additional files

Additional file 1: Forest plot of the 60-day mortality in comparison between liberal and restrictive blood transfusion strategy in sepsis or septic shock. (TIFF 10644 kb)

Additional file 2: Forest plot of the 90-day mortality in comparison between liberal and restrictive blood transfusion strategy in sepsis or septic shock. (TIFF 10644 kb)

Additional file 3: Forest plot of the ventilation use at 28 days of admission in comparison between liberal and restrictive blood transfusion strategy in sepsis or septic shock. (TIFF 10644 kb)

Additional file 4: Forest plot of the vasopressor use at 28 days of admission in comparison between liberal and restrictive blood transfusion strategy in sepsis or septic shock. (TIFF 10644 kb)

Additional file 5: Forest plot of the renal replacement therapy at 28 days of admission in comparison between liberal and restrictive blood transfusion strategy in sepsis or septic shock. (TIFF 10644 kb)

Additional file 6: Forest plot of the number of patients whom FFP was transfused during ICU stay in comparison between liberal and restrictive blood transfusion strategy in sepsis or septic shock. (TIFF 5987 kb)

Additional file 7: Forest plot of the number of patients whom platelets was transfused during ICU stay in comparison between liberal and restrictive blood transfusion strategy in sepsis or septic shock. (TIFF 5987 kb)

## Data Availability

The data and material used for this meta-analysis are contained in the references.

## Abbreviations

CI: Confidence interval
HLA: Human leukocyte antigen
ICU: Intensive care unit
OR: Odds ratio
RBC: Red blood cell
RCT: Randomized controlled trial
RRT: Renal replacement therapy
