# Supplementary figures and images for: Liberal versus restrictive red blood cell transfusion strategy in sepsis or septic shock: a systematic review and meta-analysis of randomized trials
Source: Crit Care. 2019 Jul 25;23:262. doi: 10.1186/s13054-019-2543-1 (PMC6659290; doi:10.1186/s13054-019-2543-1)

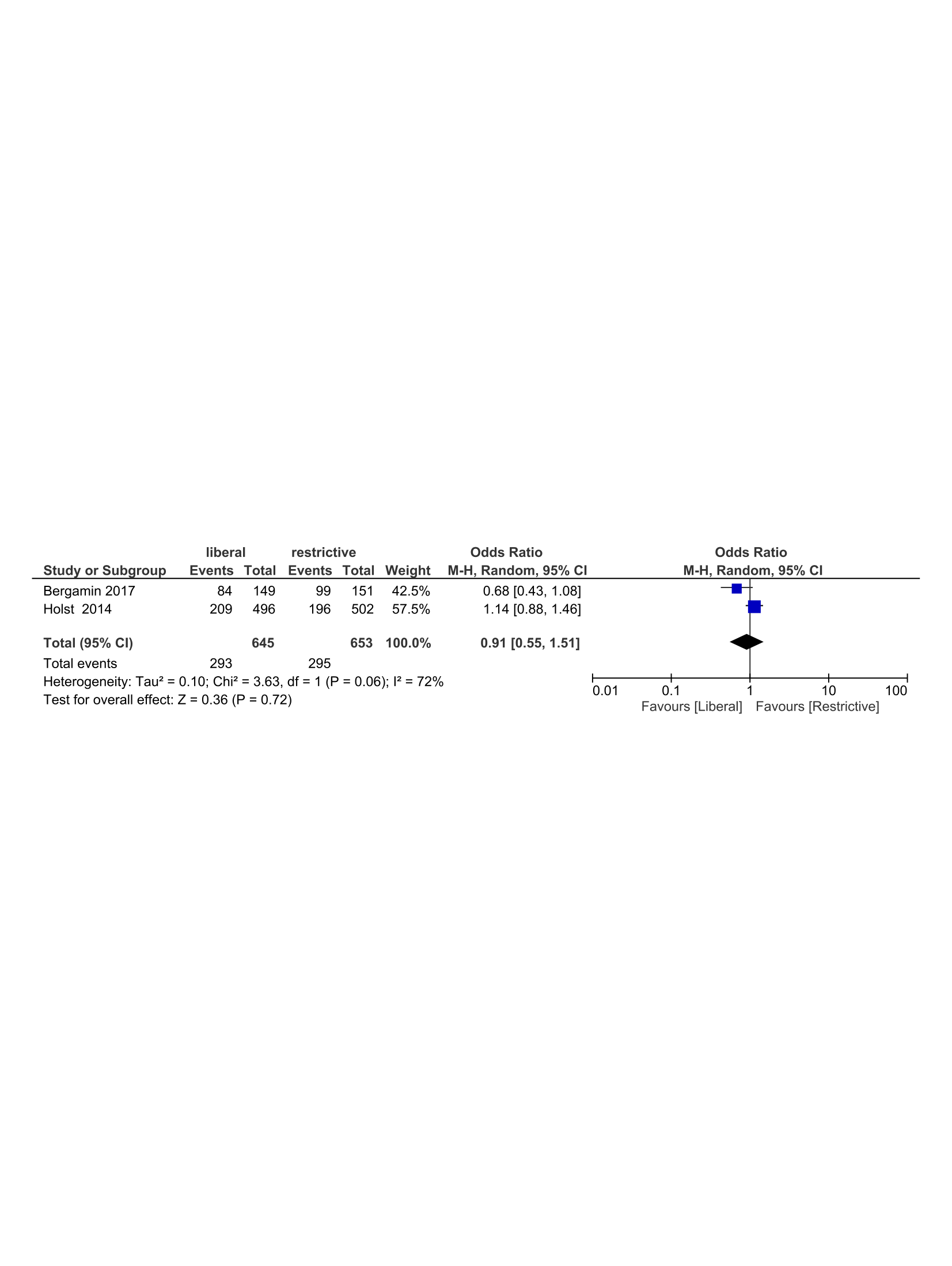

Supplement: Supplementary file 1 — Forest plot of the 60-day mortality in comparison between liberal and restrictive blood transfusion strategy in sepsis or septic shock. (TIFF 10644 kb) [file 13054_2019_2543_MOESM1_ESM.tiff]

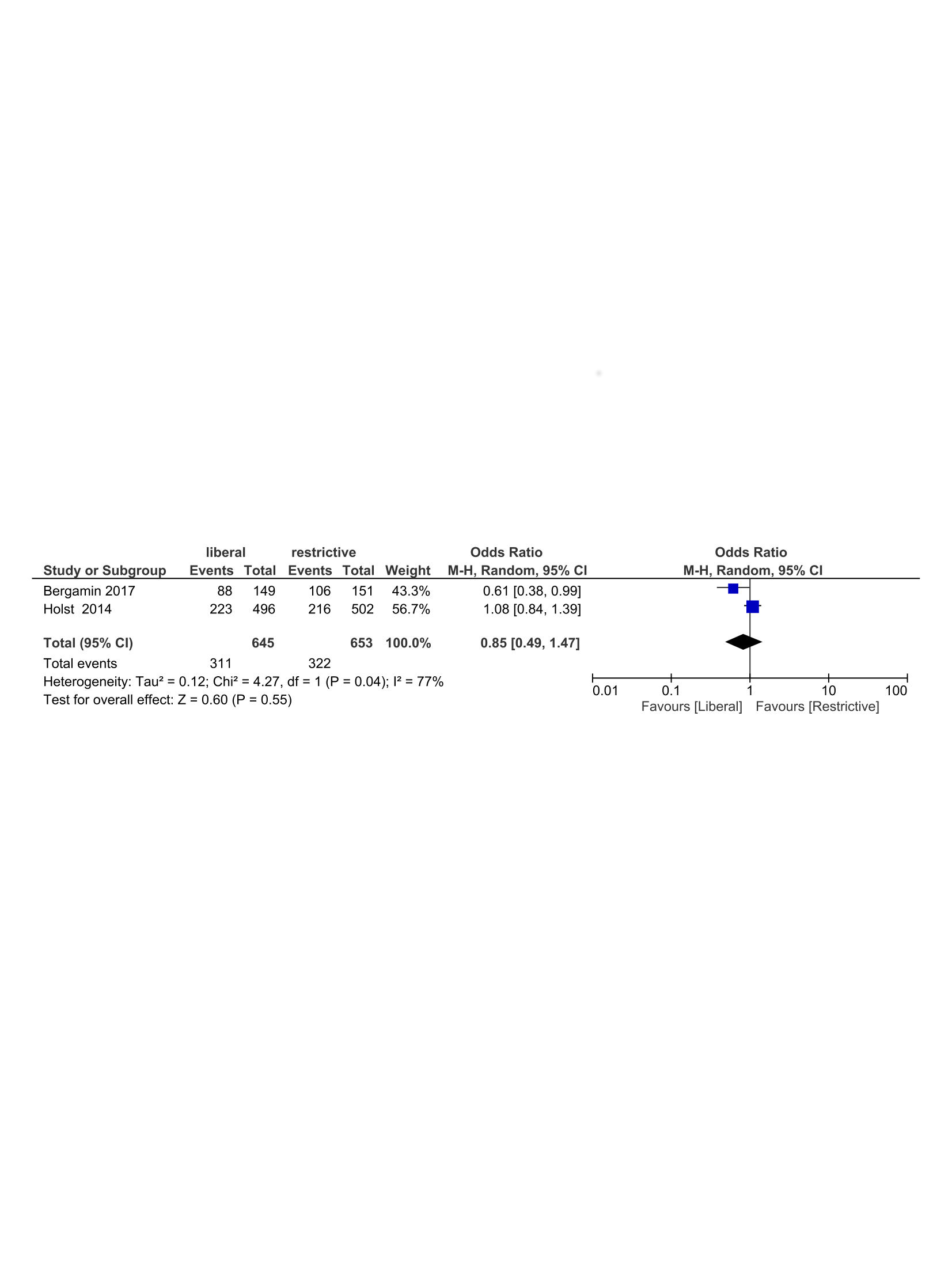

Supplement: Supplementary file 2 — Forest plot of the 90-day mortality in comparison between liberal and restrictive blood transfusion strategy in sepsis or septic shock. (TIFF 10644 kb) [file 13054_2019_2543_MOESM2_ESM.tiff]

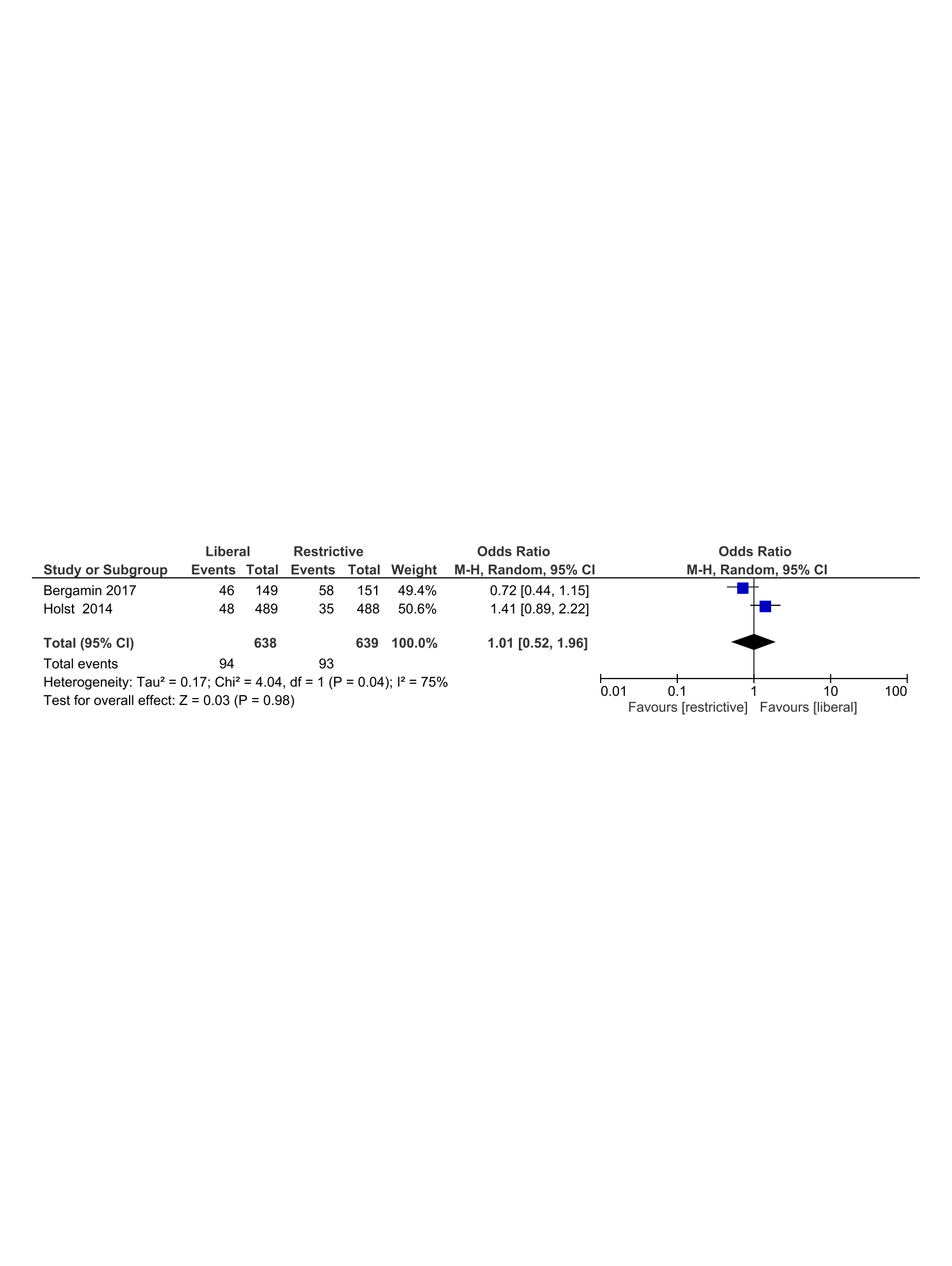

Supplement: Supplementary file 3 — Forest plot of the ventilation use at 28 days of admission in comparison between liberal and restrictive blood transfusion strategy in sepsis or septic shock. (TIFF 10644 kb) [file 13054_2019_2543_MOESM3_ESM.tiff]

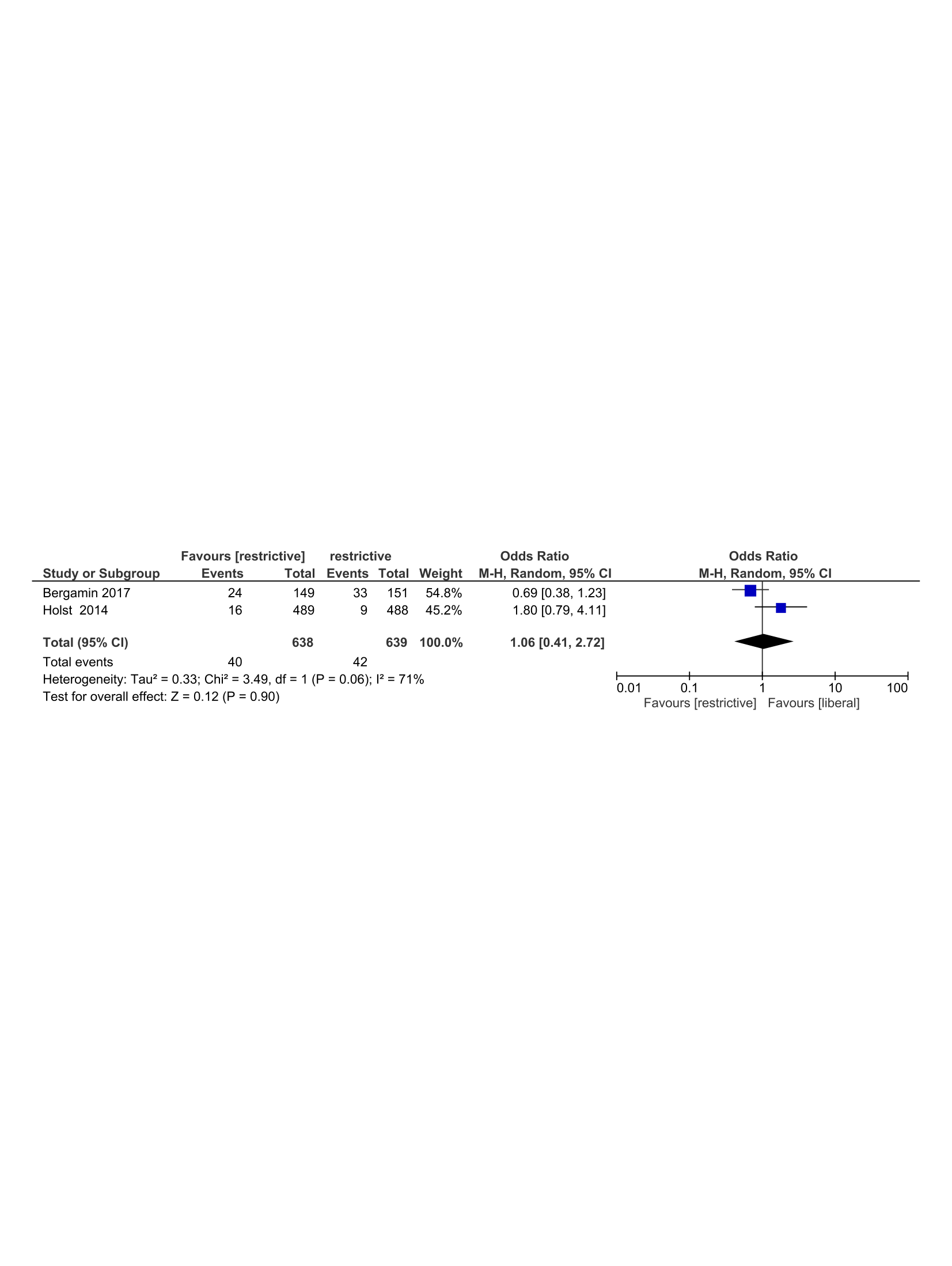

Supplement: Supplementary file 4 — Forest plot of the vasopressor use at 28 days of admission in comparison between liberal and restrictive blood transfusion strategy in sepsis or septic shock. (TIFF 10644 kb) [file 13054_2019_2543_MOESM4_ESM.tiff]

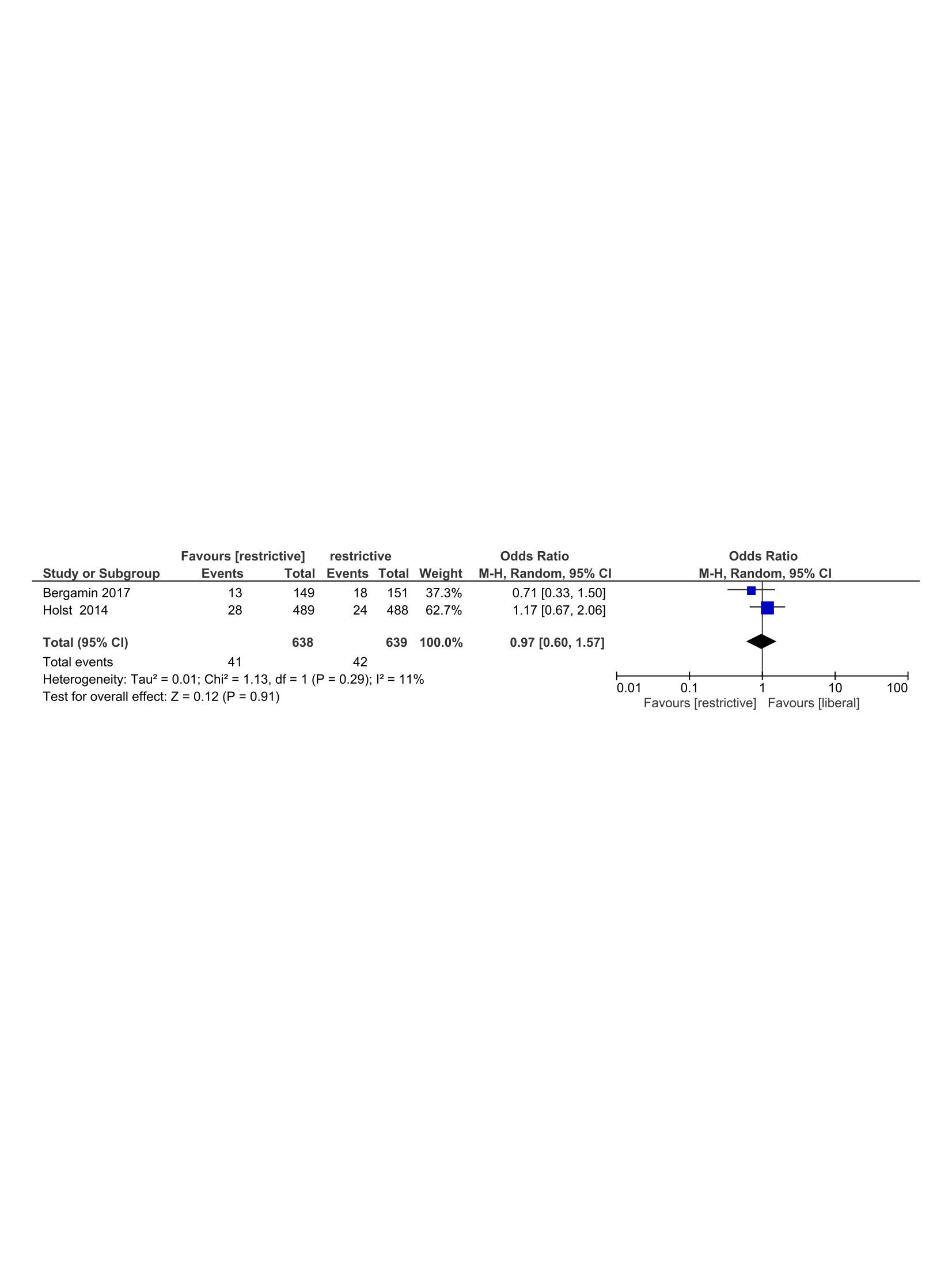

Supplement: Supplementary file 5 — Forest plot of the renal replacement therapy at 28 days of admission in comparison between liberal and restrictive blood transfusion strategy in sepsis or septic shock. (TIFF 10644 kb) [file 13054_2019_2543_MOESM5_ESM.tiff]

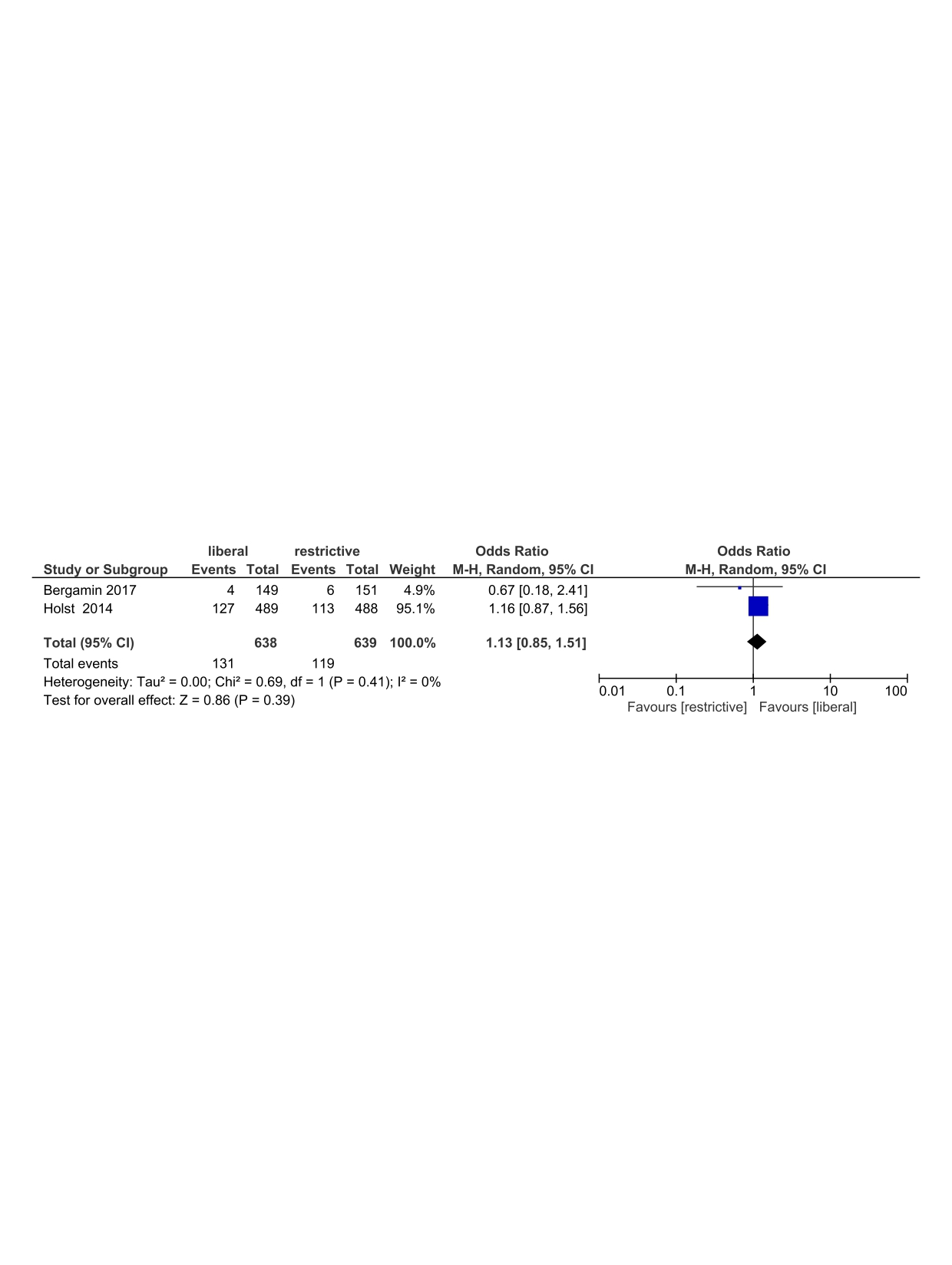

Supplement: Supplementary file 6 — Forest plot of the number of patients whom FFP was transfused during ICU stay in comparison between liberal and restrictive blood transfusion strategy in sepsis or septic shock. (TIFF 5987 kb) [file 13054_2019_2543_MOESM6_ESM.tiff]

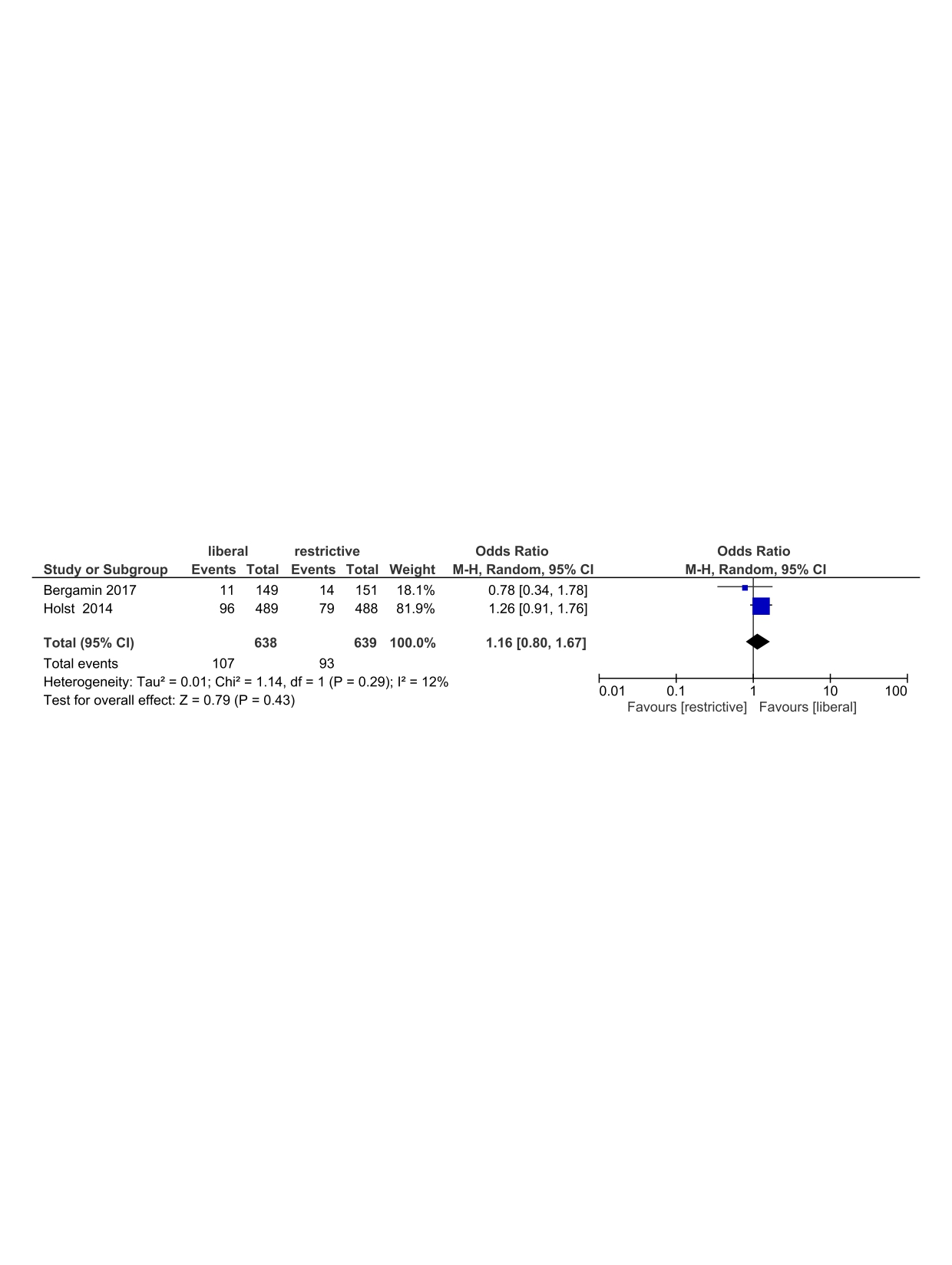

Supplement: Supplementary file 7 — Forest plot of the number of patients whom platelets was transfused during ICU stay in comparison between liberal and restrictive blood transfusion strategy in sepsis or septic shock. (TIFF 5987 kb) [file 13054_2019_2543_MOESM7_ESM.tiff]
